# Targeting Protein Kinases Degradation by PROTACs

**DOI:** 10.3389/fchem.2021.679120

**Published:** 2021-06-30

**Authors:** Fei Yu, Ming Cai, Liang Shao, Jihong Zhang

**Affiliations:** Medical School of Kunming University of Science and Technology, Kunming, China

**Keywords:** protac, protein kinase, inhibitors, degradation, anti-cancer

## Abstract

Kinase dysregulation is greatly associated with cell proliferation, migration and survival, indicating the importance of kinases as therapeutic targets for anticancer drug development. However, traditional kinase inhibitors binding to catalytic or allosteric sites are associated with significant challenges. The emergence of resistance and targeting difficult-to-degrade and multi-domain proteins are significant limiting factors affecting the efficacy of targeted anticancer drugs. The next-generation treatment approaches seem to have overcome these concerns, and the use of proteolysis targeting chimera (PROTAC) technology is one such method. PROTACs bind to proteins of interest and recruit E3 ligase for degrading the whole target protein via the ubiquitin-proteasome pathway. This review provides a detailed summary of the most recent signs of progress in PROTACs targeting different kinases, primarily focusing on new chemical entities in medicinal chemistry.

## Introduction

Cancer is a condition in which cells demonstrate abnormal growth and invasion to adjacent tissues. According to a World Health Organization (WHO) report, more than 15% of overall global deaths are due to cancer (Ward et al., 2020). Chemotherapy was first used to treat tumorous lesions in the 20th century. Several selective small-molecular anticancer drugs have been developed in the past few decades (Singh et al., 2011; Ward et al., 2020).

Small molecules have greatly improved cancer treatment, but the tumor cells are inherently resistant to certain anticancer mechanisms or acquire resistance to therapeutic agents. Alternative treatment options must be devised for patients resistant to anticancer treatment.

**FIGURE 1:**
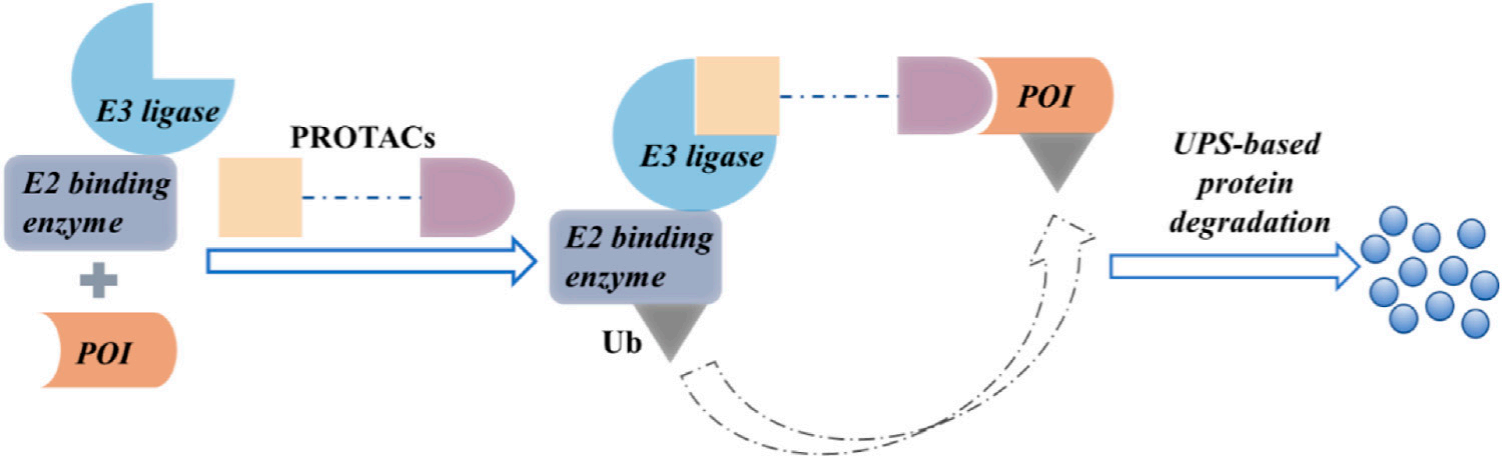
The proposed mechanism of action of PROTACs.

Recently, the use of proteolysis-targeting chimera (PROTAC) to discover and develop new drugs has been gaining significant traction. The cell cycle can be modulated by regulating the protein function. Inhibitors can modulate protein function partially, whereas PROTACs induce complete protein degradation, affecting all functions of that protein (Pettersson and Crews, 2019). A PROTAC comprises an E3 ubiquitin ligase ligand, a protein of interest (POI) ligand, and a linker. It employs heterobifunctional small molecules to recruit an E3 ubiquitin ligase and the POI (Coleman and Crews, 2018) to cause a ternary complex. The E3 ligase-mediated ubiquitination of the POI leads to proteasomal degradation (Figure 1).

PROTAC technology has been well received following the development of the first PROTAC molecule by Crews et al. (Sakamoto et al., 2001; Yin and Hu, 2020; Zhou X. et al., 2020; Martín-Acosta and Xiao, 2021; Zeng et al., 2021). ARV-110 is the first oral small-molecule PROTAC degrader that targets androgen receptors (ARs). It is being tested in clinical trials to treat metastatic castration-resistant prostate cancer. Academic and industrial research have been greatly encouraged by these advances.

In this review, we mainly summarize advances in the use of PROTACs targeting protein kinases. These findings will help drive new approaches to overcome drug resistance in cancer therapy.

## Recent Progress of PROTACs Targeting Protein Kinase

Advances in modern biological approaches have enabled the discovery of more than 500 protein kinases. These molecules affect the survival, migration, and proliferation of cells, indicating their potential as therapeutic targets for anticancer drugs. To date, more than 40 small-molecule inhibitors were approved by the US Food and Drug Administration for cancer therapy. Most of these small molecules target protein kinases, which modulate cell signaling by catalyzing the phosphorylation relevant proteins (Bedard et al., 2020).

### Proteolysis Targeting Chimeras Targeting BCR-ABL

The leukemogenic ability of BCR-ABL in chronic myeloid leukemia (CML) is well recognized, making it a potential anticancer drug target. Imatinib was the first tyrosine kinase inhibitor (TKI) targeting BCR-ABL (Schindler, 2000; Deininger et al., 2005; Reddy and Aggarwal, 2012). Subsequently, multiple ABL kinase inhibitors, including ponatinib (Gozgit et al., 2012), dasatinib (Shah et al., 2004), nilotinib (Weisberg et al., 2006), and bosutinib (Boschelli et al., 2001) have been approved for clinical use. Although TKI use has improved CML treatment outcomes, long-term therapy and drug resistance are significant challenges.

**FIGURE 2:**
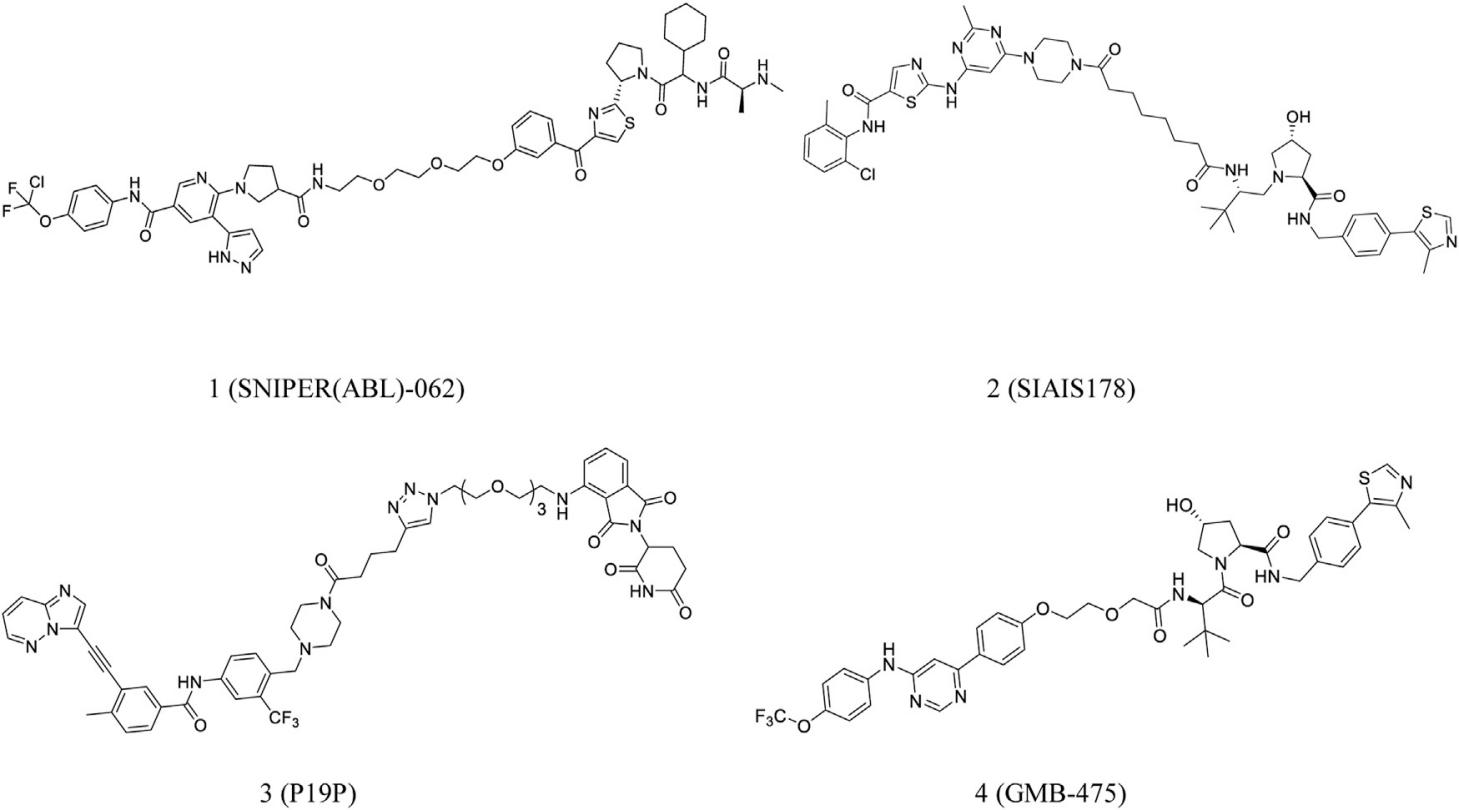
Chemical structures of PROTACs targeting BCR-ABL.

Degradation of BCR-ABL may be beneficial in CML treatment. Nagar et al. developed PROTACs comprising linkers of variable lengths in term of the co-crystal structure of the c-ABL tyrosine kinase domain-imatinib complex (Nagar et al., 2003). Lai et al. conjugated dasatinib or bosutinib to a cereblon (CRBN) E3 ubiquitin ligase ligand for successful protein degradation of BCR-ABL (Lai et al., 2016). Shimokawa et al. reported a new anticancer agent, **SNIPER(ABL)-062** (**1**, Figure 2), that degrades oncogenic proteins. The compound demonstrated strong binding affinities with cIAP1/2, ABL1 and XIAP, leading to complete BCR-ABL degradation and inhibition of the BCR-ABL-mediated signaling pathways (Shimokawa et al., 2017). Further, Demizo et al. reported a conjugate of imatinib derivative and bestatin that recruited the E3 ubiquitin ligase cIAP1 to induce BCR-ABL degradation (Demizu et al., 2016).

As mentioned earlier, a PROTAC consists of a target-protein ligand and an E3 ubiquitin ligase ligand coupled using a flexible linker (Bondeson et al., 2018; Cyrus et al., 2011). Zhao et al. speculated that a significant interaction between BCR-ABL and E3 ligase could be induced using an optimized linker. Based on this assumption, they developed **SIAIS178** (**2**, Figure 2), a strong BCR-ABL degrader. **SIAIS178** demonstrated appreciable selectivity and considerably inhibited the growth of BCR-ABL^+^ leukemic cells *in vitro*. It also induced substantial regression of K562 xenograft tumors *in vivo* (Zhao et al., 2019).

Importantly, overexpression of BCR-ABL and mutation of T315I can significantly contribute to drug resistance (Yang and Fu, 2015). However, there are no approved drugs targeting T315I-mutant cells. Yang et al. reported a series of PROTACs targeting all three binding sites of dasatinib, ponatinib, and asciminib in the BCR-ABL protein. **P19P** (**3**, Figure 2) degrades dasatinib-resistant T315I and asciminib-resistant V468F mutations in BCR-ABL and reduces the adverse effects of ponatinib (Yang et al., 2020). Moreover, **GMB-475** (**4**, Figure 2), discovered by Crews et al., induced rapid proteasomal degradation and demonstrated increased sensitivity. It also inhibited certain BCR-ABL1 kinase domain point mutations (Burslem et al., 2019).

### Proteolysis Targeting Chimeras Targeting Phosphatidylinositol 3-Kinase/Akt

Phosphatidylinositol 3-kinases (PI3Ks) are intracellular lipid kinases that play essential roles in cell survival, proliferation, growth, differentiation, and migration (Vanhaesebroeck et al., 2012; Thorpe et al., 2015). There are three classes of PI3Ks, of which Class I is widely investigated (Liu et al., 2009). Most cancer cells demonstrate hyperactivating mutations of type I PI3Ks, which drive tumor cell proliferation and survival (Sheppard et al., 2012). PI3Ks are potential anticancer targets, and agents inhibiting them are preferred in chemotherapy (Yadav et al., 2016).

**FIGURE 3:**
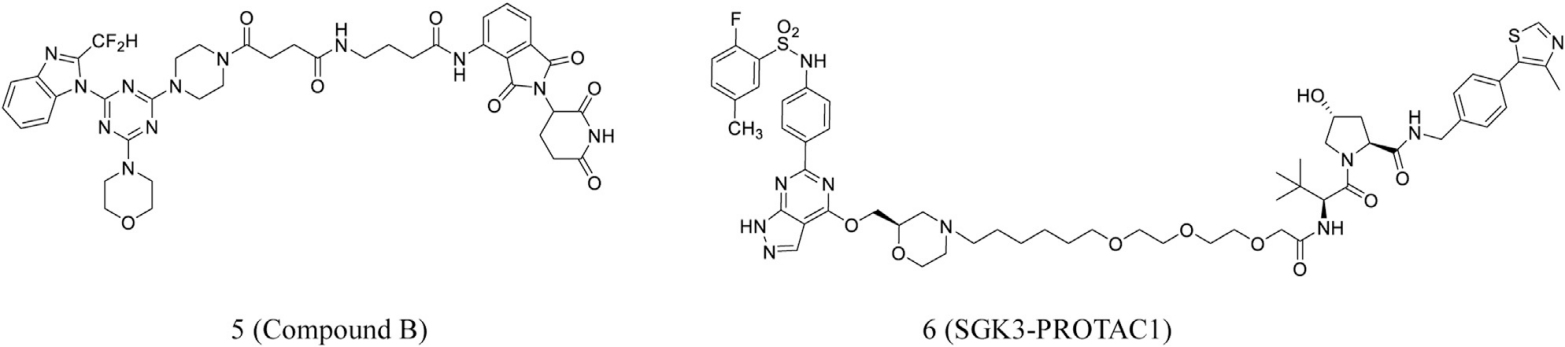
Chemical structures of PROTACs targeting PI3K/Akt.

PROTACs are also used for PI3K degradation (Hines et al., 2013; Guo et al., 2017). Li et al. developed new PI3K-PROTACs by associating pomalidomide and piperazine derivative using different linkers. Many of these compounds could significantly inhibit PI3Ka with IC_50_ values at the nanomolar level. The most potent **compound B** (**5**, Figure 3) showed an IC_50_ of 18 nM against PI3Ka. These compounds were also able to inhibit HepG2 cell proliferation (Li et al., 2018).

PI3K or Akt inhibitors activate and increase SGK3 expression in patients with ER+ breast cancer (BC) receiving long-term treatment. SGK3, instead of Akt, activates mTORC1 by inducing TSC2 phosphorylation (Bago et al., 2016). These findings implied SGK3 as an effective target to overcome resistance to PI3K/Akt inhibition in cancer treatment.

Tovell et al. reported SGK3-PROTAC1 (6, Figure 3), which selectively degrades SGK3. In HEK293 cells, the PROTAC did not degrade the SGK1 and SGK2 isoforms or other proteins. SGK3-PROTAC1, at submicromolar concentrations, induced proteasomal-mediated degradation in several cancer cell lines. It was able to cause significant degradation within 2 h (Tovell et al., 2019).

### Proteolysis Targeting Chimeras Targeting Bruton’s Tyrosine Kinase

Bruton’s tyrosine kinase (BTK) promotes B-cell growth, maturation, migration, and apoptosis (Campbell et al., 2018). Cancer, autoimmunity, or inflammation may be a result of dysregulated BTK pathways (Pal Singh et al., 2018). Studies have verified BTK as an essential target for agents against chronic lymphocytic leukemia (CLL). Research on BTKs is advancing considerably, with the BTK inhibitors ibrutinib (Advani et al., 2013), acalabrutinib (Byrd et al., 2016) and zanubrutinib (Guo et al., 2019) approved for use by the FDA. However, drug resistance and off-target effect are limitations of BTK inhibitors (Woyach et al., 2014). As such, there is an immediate need to develop drugs with new molecular entities and mechanisms targeting BTK or its mutants.

Zorba et al. synthesized 11 PROTACs and investigated their BTK-degrading abilities. They found that the length of a linker is crucial for promoting BTK and CRBN interaction (Zorba et al., 2018).

**FIGURE 4:**
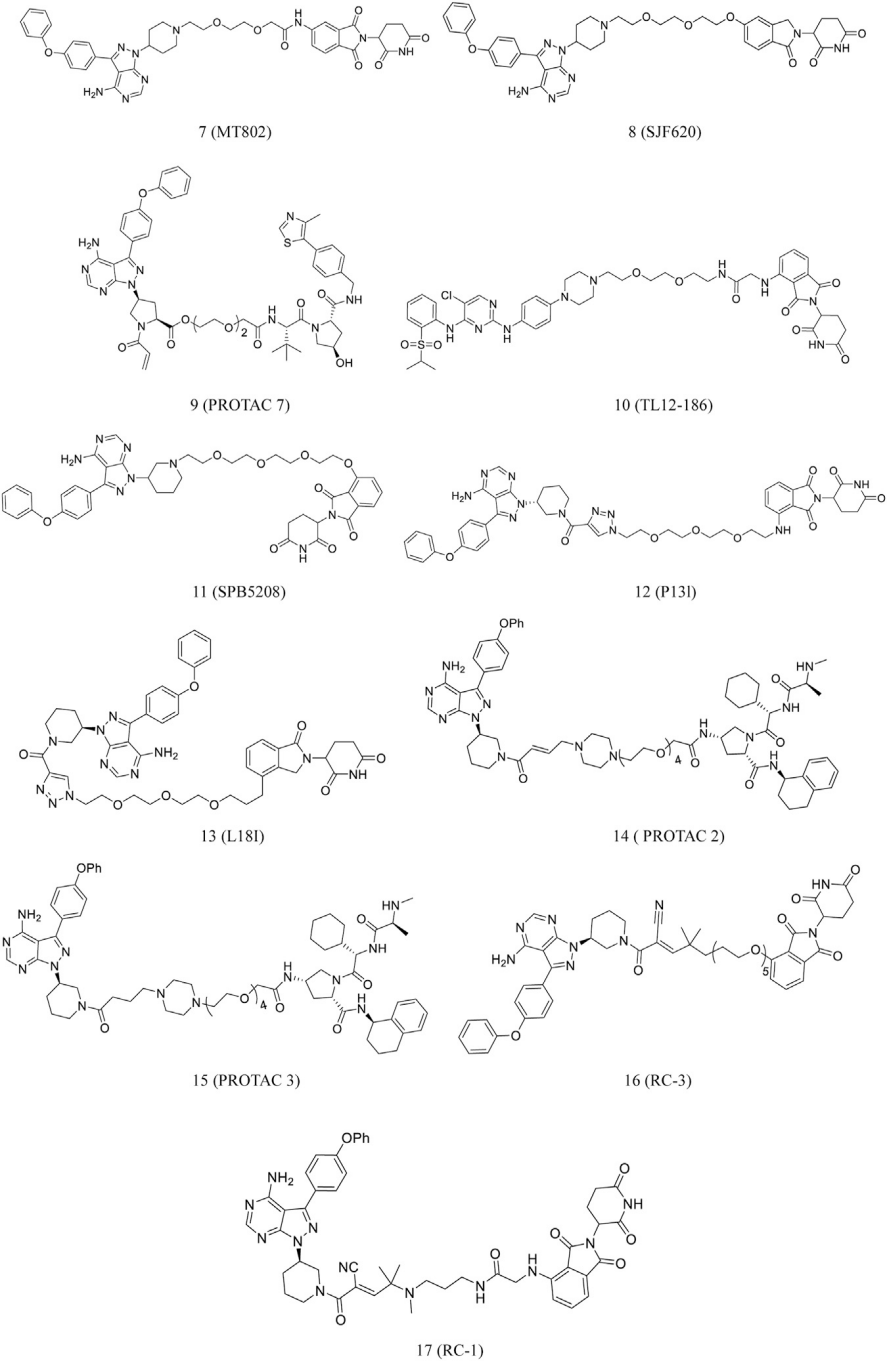
Chemical structures of PROTACs targeting BTK.

Recently, Buhimschi et al. reported the discovery of **MT802** (**7**, Figure 4), which has a BTK-specific ligand and CRBN ligand connected by a PEG linker. The compound effectively degraded BTK in cells. Compared to ibrutinib, this compound had fewer off-target kinase binding and more effective BTK degradation (Buhimschi et al., 2018).

Pharmacokinetic data on **MT802** in mice, however, demonstrated its unsuitability for further *in vivo* studies. Jaime-Figueroa et al. modified the CRBN ligand structure of **MT802** to synthesize **SJF620** (**8**, Figure 4), demonstrating a superior pharmacokinetic profile than **MT802** in mice (Jaime-Figueroa et al., 2020).

Pan et al. designed and synthesized **PROTAC 7** (**9**, Figure 4) based on their previous work. The compound significantly reduced BTK protein level and degraded the BLK protein (Chen J. et al., 2018; Xue et al., 2020).

**TL12-186** (**10**, Figure 4), a PROTAC targeting CRBN, was developed by Huang et al. The compound demonstrated >90% inhibition of 193 kinases at a tested level of 1 μM. **TL12-186** considerably downregulated the expressions of 14 out of the 7,559 identified proteins, including BTK in MOLM-14 cells, by at least 25% (Huang et al., 2018).

Liu et al. synthesized **SPB5208** (**11**, Figure 4), a BTK-degrading PROTAC, by linking ibrutinib and thalidomide. The compound reduced the activity of BTK enzyme with high selectivity and effectively suppressed the proliferation of cancer cell *in vitro*. Studies on the mechanism of action revealed that it degraded BTK JeKo-1 cells in a proteasome- and CRBN-dependent fashion. Additionally, the earlier findings suggested that this compound caused significant BTK protein degradation in ICR mice in a manner of dose-dependent (Liu et al., 2020).

Ibrutinib was conjugated to pomalidomide to synthesize P13l (12. Figure 4). The compound developed by Sun et al. induced 73% degradation of BTK in RAMOS cells at 10 nM. BTK degradation began at about 4 h after treatment with PI3I and was completed by 24 h. In addition, the compound could effectively degrade BTK in MCL and MM cell lines (Sun et al., 2018). Mantle cell lymphoma (MCL) accounts for more than 6–8% of all non-Hodgkin lymphomas (NHLs) worldwide (Sun et al., 2018). The tumor cells with drug-resistant were obtained from patients with MCL in the course of ibrutinib treatment. The relapse C481S missense BTK mutation could have contributed to this resistance (Chiron et al., 2014). During the growth suppression of diffuse large B-cell lymphoma (DLBCL), the BTK C481S mutant lead to resistance to ibrutinib (Sun et al., 2018). Based on these findings, Sun et al. developed **L18I** (**13**, Figure 4), which could induce the degradation of ibrutinib-resistant C481S BTK in HBL-1 cells at 30 nM. Excitingly, **L18I** also caused an apparent antitumor effect in mice culture with C481S BTK HBL-1 cells (Sun et al., 2019).

To date, most reported PROTACs reported operate through covalent or noncovalent binding. Irreversible covalent inhibitors have strong target affinities and high target occupancies and have been successful in the clinical setting However, irreversible bindings may reduce the potency by negating the catalytic nature of the PROTAC activity (Gabizon et al., 2020).

Dittus et al. selected the covalent inhibitor ibrutinib and a reversibly binding analog to develop **PROTAC 2** (**14**, Figure 4) and **PROTAC 3** (**15**, Figure 4), which were investigated for their BTK degradation capabilities (Dittus et al., 2017). **PROTAC 2** did not degrade the covalently bound targets, whereas **PROTAC 3** degraded its target protein. This finding highlights the importance of catalysis for successful PROTAC-mediated degradation (Tinworth et al., 2019).

Is there an effective way to bind PROTACs covalently to the target, and at certain conditions, is this covalent bond reversible? Theoretically, the selectivity, enhanced potency, and long duration of action that accompany covalent bond formation can promote reversible covalent PROTACs (Serafimova et al., 2012; Bradshaw et al., 2015). Moreover, reversible covalent PROTACs could combine the advantages of covalent binding, including the selectivity and increased potency, while keeping the reversibility, which is necessary for catalytic properties of chemical reactions of a PROTAC’s efficacy.

Gabizon et al. tested this hypothesis by designing acrylamide- and cyanoacrylamide-based reversible covalent PROTACs. BTK was selected as the target, and irreversible covalent, noncovalent, and reversible covalent PROTACs were systematically analyzed. Cyanoacrylamide-containing PROTACs were reported to be much more potent than acrylamide analogs and equivalent noncovalent PROTACs. Based on these data, a highly potent, selective, reversible covalent **RC-3** (**16**, Figure 4) was synthesized**.** The compound demonstrated increased selectivity for the BTK protein than its noncovalent and irreversible covalent counterparts (Gabizon et al., 2020). These findings suggested the potential use of PROTACs as a plethora of challenging targets.

PROTACs are high-molecular-weight compounds, resulting in poor membrane permeabilities and, subsequently, low intracellular concentrations compared with other drugs. As such, they demonstrate low target occupancy and depend heavily on substoichiometric protein degradation for therapeutic efficacy (Guo et al., 2020).

Guo et al. discovered that a cyanoacrylamide-based reversible covalent binder to BTK significantly increased target engagement and the accumulation of drug in cells (Guo et al., 2020). It is known that cyanoacrylamides reversibly react with thiols with millimolar dissociation equilibrium constants (Kd) and rapid kinetics (Krishnan et al., 2014; Bradshaw et al., 2015; Jiang et al., 2017). Based on these findings, they developed **RC-1** (**17**, Figure 4), a reversible covalent BTK PROTAC, which was the most potent BTK degrader reported at that time (Zorba et al., 2018; Tinworth et al., 2019). The compound effectively degraded BTK at 8–40 nM. It also induced BTK degradation in MOLM-14 cells and also degraded BTK regardless of its mutation status, which was surprising. This reversible covalent strategy can be generalized to other PROTACs to develop a new approach to enhance the efficacy of a PROTAC (Guo et al., 2020).

### Proteolysis Targeting Chimeras Targeting Receptor Tyrosine Kinases

Following the identification of the cDNA encoding the epidermal growth factor receptor, studies were conducted to understand critical roles of receptor tyrosine kinases (RTK) in signaling pathways governing fundamental cellular processes and processes that regulate intercellular communications.

Dysregulation of protein kinases is well recognized in cancer cells (Gossage and Eisen, 2010). EGFR, as a kind of RTK, is critically associated with the regulation of cell apoptosis, proliferation, metabolism, and survival (Yarden and Sliwkowski, 2001). Overexpression and activating mutations of EGFR are related to several cancer types. Unregulated activation of EGFR leads to abnormal cell growth and downstream signaling. These adverse effect may lead to the endocytosis of EGFR signal and intracellular terminal transport (Rosell et al., 2009; Yewale et al., 2013).

Recently, some therapeutic agents targeting EGFR with small molecules and antibodies were developed (Maennling et al., 2019). This protein has been extensively investigated in oncogenic and normal signaling, and EGFR-based anticancer drugs are commonly used therapeutically (Ayati et al., 2020). To date, over ten EGFR inhibitors have been given to patients with non-small-cell lung cancer by FDA, but the EGFR-mutant variants may compromise drug efficacy (Ayati et al., 2020). Unlike kinase activity inhibition, EGFR degradation results in complete, lasting downstream signal inactivation. PROTACs degraded several RTKs, including c-Met, HER2, EGFR, and multiple mutants of EGFR and c-Met.

**FIGURE 5:**
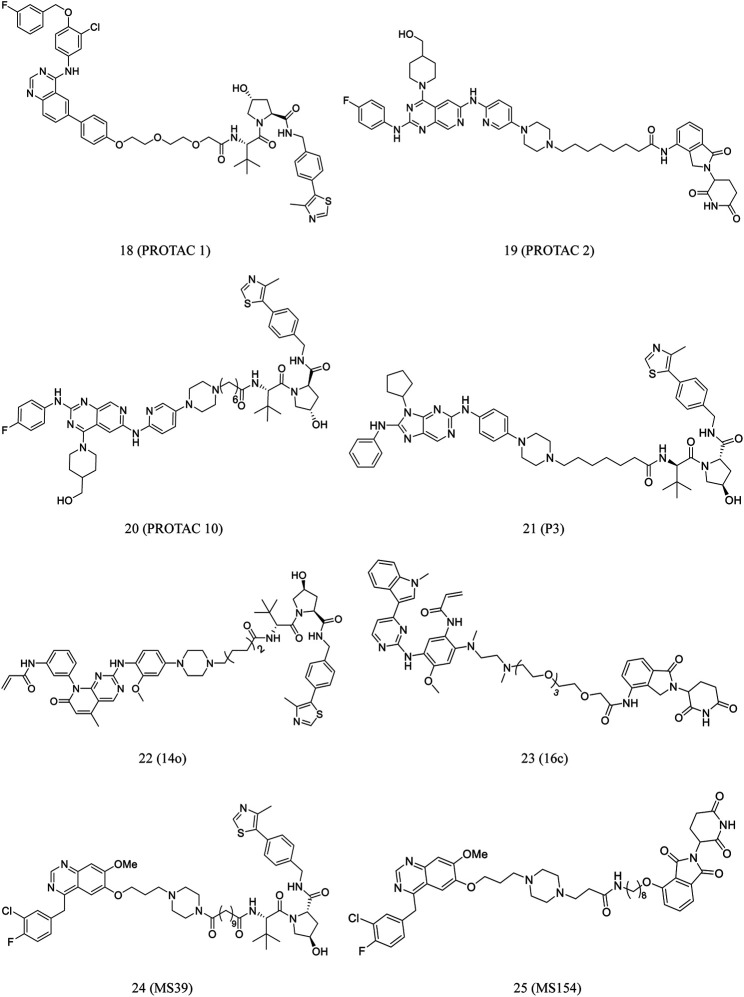
Chemical structures of PROTACs targeting RTK.

Konecny et al. synthesized **PROTAC 1** by conjugating lapatinib, an EGFR-binding element, to an E3 ligase binding ligand (**18**, Figure 5). At low nanomolar concentrations, the compound demonstrated cell-membrane penetration and EGFR degradation (Konecny et al., 2006; Buckley et al., 2012; Burslem et al., 2018).

A new series of EGFR degraders containing the pyrido[3,4-*d*]pyrimidine moiety were proposed and developed by the Zhang et al. In HCC827 cells, **PROTACs 2** (**19**, Figure 5) and **10** (**20**, Figure 5) effectively degraded EGFR. Furthermore, they significantly induced HCC827 cell apoptosis (Zhang H. et al., 2020).

Gefitinib, a first-generation EGFR TKI, targets mutated and overactive EGFR and provides considerable clinical benefits (Mok et al., 2009). However, acquired resistance is known to develop following short-term treatment with the compound (Yu et al., 2013). To overcome T790M EGFR mutation-induced drug resistance, second and third generation EGFR inhibitors were developed (Sos et al., 2010). Osimertinib, a third-generation EGFR inhibitor, was approved by FDA and used in the patients with metastatic NSCLC in 2015. Regarding the mechanism of drug action, the acrylamide moiety in osimertinib undergoes a Michael addition reaction with the Cys797 residue to get inhibitory activity against the EGFR^T790M^ mutants (Finlay et al., 2014; Jänne et al., 2015). Unfortunately, newly acquired drug resistance has been identified (Thress et al., 2015; Bersanelli et al., 2016; Zheng et al., 2017; Chen L. et al., 2018).

EGFR^L858R/T790M^ serves as an ideal and relatively safe target for PROTACs because it is selectively expressed in tumor cells with acquired drug resistance, but it can be found in normal cells. Multiple selective EGFRL858R/T790M mutant PROTAC degraders were proposed and developed by Zhang et al. Of these degraders, **14o** (**22**, Figure 5) is the most potential as PROTAC to effectively and selectively degrade EGFR^L858R/T790M^ (Zhang X. et al., 2020).

Zhao et al. reported a series of EGFR-targeting small-molecule PROTACs, which demonstrated potent efficacies. Of these PROTACs, **P3** (**21**, Figure 5) displayed intense antiproliferative activity against the cell lines of H1975 and HCC827. In this process, the IC_50_ values of **P3** (**21**, Figure 5) against H1975 and HCC827 was 203.01 and 0.83 nM, respectively. Moreover, It was reported that **P3** treatment significantly degraded EGFR^L858R/T790M^ and EGFR^del19^ (Zhao et al., 2020).

He et al. developed a class of small-molecule EGFR degraders by conjugating lenalidomide with osimertinib through different linkers. Compound **16c** (**23**, Figure 5) effectively degraded EGFR in PC9 cells, with the maximum degradation rate at 68%. Furthermore, it promoted PC9 cell apoptosis and arrest in the G0/G1 phase (He et al., 2020).

Cheng et al. developed a novel gefitinib-based VHL-recruiting EGFR degrader (**MS39**, **24**, Figure 5) and a first-in-class gefitinib-based CRBN-recruiting EGFR degrader (**MS154**, **25**, Figure 5). Both compounds selectively induced degradation of mutant EGFR and downstream signaling inhibition in cells, whereas no apparent effect was noted on the wild-type protein (Cheng et al., 2020).

### Proteolysis Targeting Chimeras Targeting Focal Adhesion Kinase

Focal adhesion kinase (FAK), a cytoplasmic tyrosine kinase that was first reported in 1992 (Mitra et al., 2005). FAK promotes tumor growth, invasion, and metastasis through kinase-dependent manner. It was found that FAK acts as a kinase and serves as a scaffold for some signal proteins (Hanks et al., 2003; Parsons, 2003; Sulzmaier et al., 2014; Lee et al., 2015; Aboubakar Nana et al., 2019). It was suggested that the increased FAK expression and activity in the primary and metastatic cancer tissues played a critical role in the poor survival of patients (Sulzmaier et al., 2014).

FAK inhibition in metastatic cancers can potentially prevent tumor progression. FAK inhibitors regulate cancer cell angiogenesis, invasion, and migration, and ECs in tumor angiogenesis. Several studies have been conducted to investigate the clinical effect of small-molecule FAK inhibitors (Chambers et al., 2002; Parsons et al., 2008; Lu and Sun, 2020).

Unfortunately, FAK has both kinase-dependent and kinase-independent functions, implying that FAK inhibitors can antagonize its kinase-dependent functions but not kinase-independent functions. However, PROTACs targeting FAK can inhibit both kinase-dependent and kinase-independent functions of the kinase (Gao et al., 2019).

**FIGURE 6:**
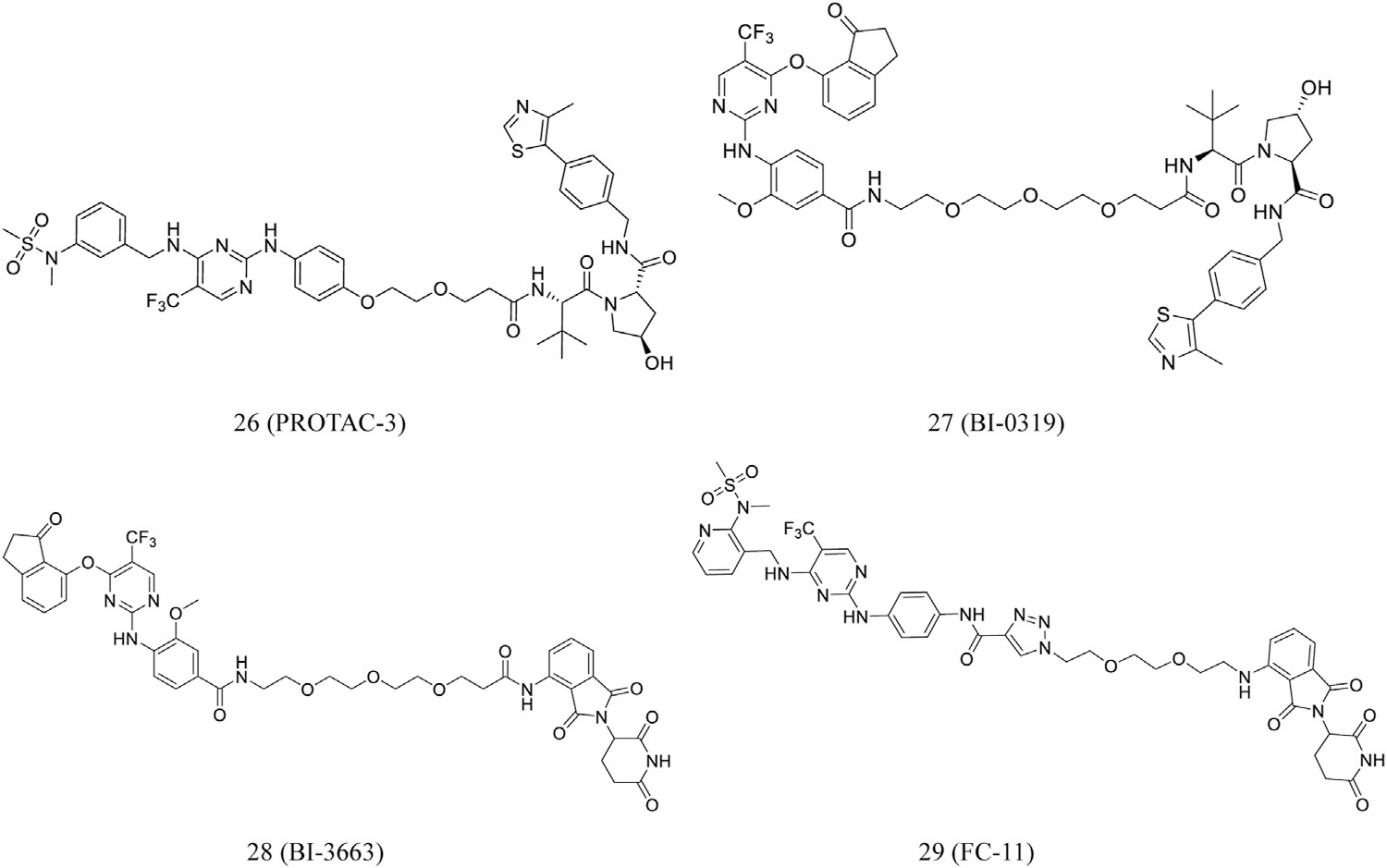
Chemical structures of PROTACs targeting FAK.

According to clinical FAK inhibitor defactinib, Luzzio et al. designed certain FAK-targeting PROTACs. Guided by earlier investigations on the SAR, the aminomethyl part of defactinib was used as a linker incorporation (Luzzio et al., 2017). The most promising degrader, **PROTAC-3** (**26**, Figure 6), is superior to defactinib in inhibiting FAK signaling and cell migration and invasion in human triple-negative breast cancer cells. Moreover, **PROTAC-3** was reported to improve selectivity over defactinib, as it only binds to FAK with less than 1% of the control compound remaining (Cromm et al., 2018).

Human hepatocellular carcinoma (HCC) cells overexpress focal adhesion tyrosine kinase (PTK2). Highly selective PROTACs targeting PTK2 degradation were developed by Popow et al. Structure-guided conjugation of a highly selective PTK2 inhibitor to either a CRBN or VHL ligand by polyethylene glycol linkers led to the development of selective PTK2 degraders **BI-0319** (**27**, Figure 6) and **BI-3663** (**28**, Figure 6), respectively. Both compounds could selectively degrade the PTK2 protein. However, further research suggested that **BI-3663-** and **BI-0319**-mediated PTK2 depletion is unlikely sufficient to investigate the inhibition effect of kinase activity under the condition of affecting proliferation *in vitro* (Popow et al., 2019).

Based on the FAK inhibitor and CRBN E3 ligand, Gao et al. developed a FAK PROTAC library. **FC-11** (**29**, Figure 6), a novel FAK-targeting PROTAC, rapidly degraded FAK after 8 h of treatment in various cell lines. Furthermore, the FAK proteins can be completely recovered after being washed away by PROTAC molecules (Gao et al., 2019).

### Proteolysis Targeting Chimeras Targeting Cyclin-dependent Kinase

The previous study reported that in the cyclin dependent kinases (CDKs) family, CDK1-4, CDK11, and CDK6 regulate the cell cycle, whereas CDK7-9 mainly regulated transcription (Bellan et al., 2004). In the earlier studies, clinical trials have been investigating the effects of small-molecule CDK inhibitors on various cancers, such as acute myeloid leukemia (AML), BC, NSCLC, and prostate cancer (PC) (Malumbres et al., 2008; Heathcote et al., 2010; Parry et al., 2010; Siemeister et al., 2012; Cicenas et al., 2014; Heptinstall et al., 2018). In 2015, palbociclib, the first selective CDK4/6 inhibitor, was approved for the treatment of metastatic BC by the FDA (Bedard et al., 2020).

Furthermore, terminally differentiated cells demonstrate increased CDK9 expression (Bagella et al., 1998). Some studies found that selective CDK9 inhibitors had the potential to treat a variety of human diseases, including cancer. A lot of evidence suggested that CDK9 is a potential tumor therapeutic target. The previous studies illustrated that the adverse effects and toxicities limited the wide application of CDK9 inhibitors in clinic (Diab et al., 2007; Tong et al., 2010). Therefore, there is a need to develop feasible treatment strategies for CDK9-induced malignant tumors (Wu et al., 2020).

**FIGURE 7:**
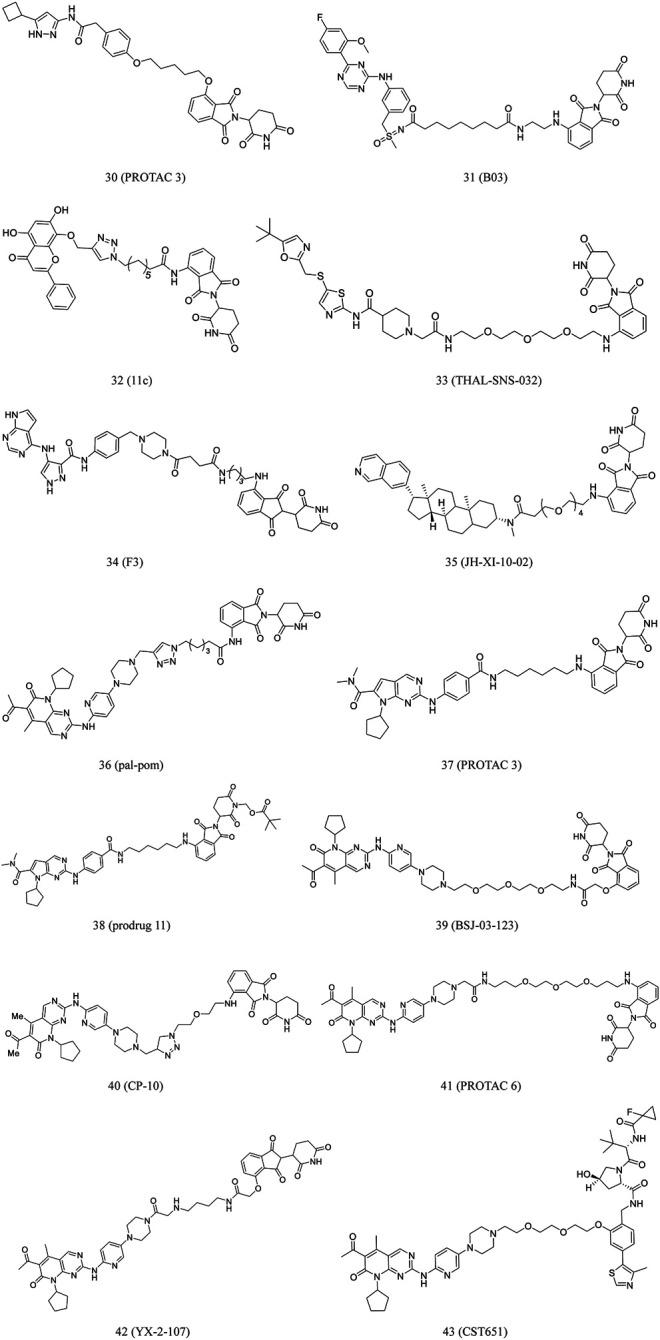
Chemical structures of PROTACs targeting CDK.

**PROTAC 3** (**30**, Figure 7) was developed by conjugating an aminopyrazole derivative with thalidomide. CDK9 can be degraded by this compound in a dose-dependent manner. The previous study found that in CDK family members, this compound only selectively degrades CDK9 in HCT116 cells (Robb et al., 2017).

Qiu et al. converted the CDK9 inhibitor BAY-1143572 into some PROTACs, which induced CDK9 degradation in AML cells at low nanomolar concentrations. The most potent compound, **B03** (**31**, Figure 7), inhibited cell growth much more effectively than BAY-1143572 alone, with no evident inhibition of other kinases. Moreover, it could induce CDK9 degradation *in vivo* (Qiu et al., 2021).

Wogonin, as a product obtained from *Scutellaria baicalensis*, is a potent and selective inhibitor of CDK9 (IC_50_ = 0.19 μM). *In vivo* studies found that wogonin induced apoptosis and suppressed the growth of human cancer xenografts (Polier et al., 2011; Ding et al., 2012). By using this scaffold, Bian et al. discovered CDK9-targeting PROTAC **11c** (**32**, Figure 7) by recruiting ubiquitin E3 ligase and CRBN (Bian et al., 2018). Western blot results revealed that **11c** selectively downregulated the intracellular CDK9 level in a concentration-dependent manner (Bian et al., 2018). The researchers also found that **11c** has the ability to promote proteasome- and CRBN-dependent degradation. This study contributed to the development of PROTACs derived from natural products for cancer treatment.

Olson et al. developed **THAL-SNS-032** (**33**, Figure 7) by conjugating selective ATP competitive CDK9 inhibitor and selective CDK9 degradation agent composed of thalidomide. Surprisingly, it was found that the compound induced highly selective and rapid degradation of CDK9 without affecting the levels of other related targets (Olson et al., 2018).

Zhou et al. have been investigating small-molecule degraders targeting CDKs. They identified **F3** (**34**, Figure 7), a dual degrader, that could potentially degrade both CDK9 and CDK2. It inhibited cell proliferation through effective blockade of the S and G2/M phases of PC-3 cells. Furthermore, it degraded CDK2/9 activities in various cancer cell lines, suggesting its anticancer potential (Zhou F. et al., 2020).

CDK8 modulates gene transcription and, therefore, plays an important role in the function of stem cells, inflammation and immune response (Carlsten et al., 2013; Allen and Taatjes, 2015). In melanoma tissues, CDK8 was overexpressed, indicating its essential role in cell proliferation (Kapoor et al., 2010). CDK8 knockdown suppressed cell growth and promoted cell cycle arrest, and hence apoptosis, in tumor models (Firestein et al., 2008). These findings indicate that small-molecule-induced inhibition of CDK may be a promising therapeutic strategy for cancer.

Hatcher et al. conducted a study to investigated highly selective CDK8 inhibitor, **JH-VIII-49.** The compound is an analog of cortistatin A with slightly reduced efficacy against CDK8 in biochemical and cellular tests. According to this scaffold, they further developed **JH-XI-10-02** (**35**, Figure 7), a bivalent degrader that can induce significant degradation of CDK8 via recruiting the E3 ligase in Jurkat cells (Hatcher et al., 2018).

In cancer treatment, CDK4/6 are attractive targets, and palbociclib, ribociclib, and abemaciclib, are dual inhibitors. The FDA approved CDK4/6 inhibitors for advanced or metastatic BC to enhance. progression-free survival by FDA (Bedard et al., 2020).

A series of imide-based degrader molecules with the potential to inhibit CDK4/6-induced cellular effects were developed by Jiang et al. These compounds successfully reduced pRB levels, promoted G1 arrest, and induced antiproliferative activity in the treated cells (Jiang et al., 2019). Similarly, Zhao and Burgess developed CDK4/6-targeting PROTACs synthesized by conjugating pomalidomide to palbociclib or ribociclib. The most potent compound, **pal-pom** (**36**, Figure 7), was remarkably potent than other CDK8-9-targeting compounds (Zhao and Burgess, 2019).

CDK2 overexpression and its complex with cyclins are positively associated with dysregulation of the cell cycle (Smalley et al., 2008). CDK 2/4/6-targeting inhibitors were regarded as a feasible chemotherapeutic regimen.

According to a ribociclib derivative and a CRBN ligand, Wei et al. developed **PROTAC 3** (**37**, Figure 7). This compound could simultaneously and effectively degrade CDK 2/4/6 and their complexes in melanomas. Furthermore, in various types of cancer cells, it can quickly start the cell cycle and cause apoptosis. The structure of **PROTAC 3** was modified to obtain its prodrug 11 (**38**, Figure 7), which demonstrated good oral bioavailability in animal studies (Wei et al., 2021).

The FDA has approved several CDK4 and CDK6 inhibitors for effective treatment of BC. Recent reports have highlighted the central role of CDK6 in transcriptional regulation (Kollmann et al., 2016). However, it is challenging to investigate its kinase-independent functions due to lack of selectivity. Brand et al. reported **BSJ-03-123** (**39**, Figure 7), a degradation agent based on phthalimide (Su et al., 2019). It was found that this degradation agent can achieve proteomic selectivity of CDK6 degradation by using protein interface determinants (Brand et al., 2019).

Su et al. developed a compound library of CDK6 degraders by linking palbociclib (CDK inhibitor) and pomalidomide (E3 ligase CRBN recruiter). These PROTAC degraders strongly inhibited proliferation of hematopoietic cancer cells and degraded copy-amplified or mutated CDK6.

Moreover, to understand the binding mechanism between PROTAC and target, researchers evaluated binding affinity, spatial orientation, and linker length. The most potent PROTAC **CP-10** (**40**, Figure 7) prevented the proliferation of several hematopoietic cancer cells. Additionally, it was reported that this compound can degrade CDK6 from mutation and overexpression (Su et al., 2019).

In contrast to findings of Su et al. (Su et al., 2019), which suggested that VHL or IAP PROTACs could not degrade CDK4 and CDK6, Anderson et al. observed that palbociclib-based PROTACs recruiting different E3 ligases demonstrated preferential CDK6 vs. CDK4 degradation selectivity (Anderson et al., 2020).

Rana et al. reported the development of five PROTACs synthesized by conjugating palbociclib to pomalidomide using flexible linkers of varying lengths and compositions. **PROTAC 6** (**41**, Figure 7) demonstrated potent, selective degradation of CDK6, sparing other CDKs (Rana et al., 2019).

CDK6 silencing seems to be more effective than palbociclib in suppressing Ph^+^ ALL in mice models. De Domici et al. developed PROTACs that induced CDK6 degradation *in vitro*. **YX-2-107** (**42**, Figure 7) could promote the rapid and preferential degradation of CDK6 over CDK4 in Ph^+^ ALL cells, as well as inhibit the activity of phospho-RB, FOXM1, and proliferation (De Dominici et al., 2020).

VHL-based PROTACs, developed by Steinebach et al., are highly active, selective CDK-6 inhibitor with demonstrated efficacy in several human and murine cancer cells. Of these PROTACs, **CST651** (**43**, Figure 7) selectively induced CDK6 degradation, and in this process, when the effective concentration was 0.1 μM, it did not affect the viability of MDA-MB-231 cells. The compound demonstrated effective and durable degradation of human and mouse cells. It also suppressed the proliferation of myeloma, leukemia, and BC cells. Moreover, **CST651** could significantly impair cell migration and reduce wound healing by 29% (Steinebach et al., 2020).

### Proteolysis Targeting Chimeras Targeting MEK1/2

The previous study suggested that the RAS-RAF-MEK-ERK was involved in several cellular processes, such as proliferation, differentiation, apoptosis, migration, and metabolism (Scholl et al., 2007; Roskoski, 2012). Additionally, MEK1 and MEK2 played important roles in regulating the activity of ERK. ERK signaling hyperactivation induced by MEK1 or MEK2 receptor mutations is related to human cancers. To date, four drugs targeting MEK1/2, selumetinib, binimetinib, cobimetinib, trametinib, were approved by the FDA for treatment of cancer. However, drug resistance to MEK1/2 inhibitors in patients gradually appeared in clinical use, implying the need for new therapeutic strategies.

**FIGURE 8:**
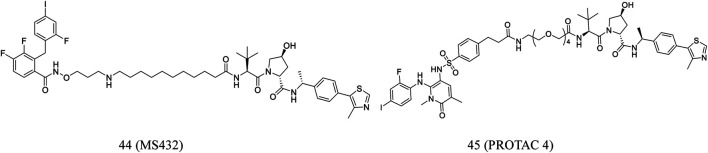
Chemical structures of PROTACs targeting MEK.

Wei et al. reported **MS432** (**44**, Figure 8), a first-in-class PROTAC of MEK1/2, developed by conjugated the MEK1/2 inhibitor PD0325901. The compound effectively degraded MEK1 and MEK2 in a concentration- and time-dependent and durable manner and prevented the phosphorylation of ERK in cellular experiments. In addition, the compound was well exposed in the plasma of mice, indicating its suitability for *in vivo* efficacy studies (Wei et al., 2019).

Vollmer et al. designed **PROTAC 4** (**45**, Figure 8) according to PEG linker and allosteric MEK inhibitors. Although **PROTAC 4** is less effective in inhibiting ERK1/2 phosphorylation, it is more potent in inhibiting A375 cell proliferation (Vollmer et al., 2020).

## Perspective

PROTACS can disrupt enzymatic and non-enzymatic functions of target proteins, demonstrating a novel approach to address acquired drug resistance. The aforementioned findings indicate the significant progress of PROTAC technology in recent years.

This strategy is particularly suitable for protein kinases. Abnormal functions of protein kinases have been closely related to cancers, highlighting the potential importance of these kinases as therapeutic targets for anti-cancer drug development. Nowadays, both multi-targeted and highly selective kinase inhibitors are used for the treatment of cancers. However, the mutant kinases and emergence of drug resistance are still major factors limiting the efficacy of kinase inhibitors.

In the past few decades, numerous ligands selectively targeting kinases as competitive or allosteric inhibitors have been developed, which provides an appropriate basis for PROTAC molecular designing. In addition, the kinase degradation mediated by PROTACs can also be used to regulate phosphorylation, expanding the applications of PROTACs.

Although the benefits of PROTACs are significant, they still have certain limitations, which need further investigation. Further insights into the action mechanisms involved in these challenges will enable scientists to address the limitations and develop next-generation PROTACs. The emergence of novel PROTACs, including reversible covalent protein-degraders, indicates their potential to be used as antitumor therapeutic agents targeting protein kinases in addition to small-molecule inhibitors and monoclonal antibodies.
